# Comparative Genome Analysis of Piscine *Vibrio vulnificus*: Virulence-Associated Metabolic Pathways

**DOI:** 10.3390/microorganisms12122518

**Published:** 2024-12-06

**Authors:** Thararat Phurahong, Patcharee Soonson, Jumroensri Thawonsuwan, Varin Tanasomwang, Nontawith Areechon, Teerasak E-kobon, Sasimanas Unajak

**Affiliations:** 1 Department of Biochemistry, Faculty of Science, Kasetsart University, 50 Ngam Wong Wan, Chatuchak, Bangkok 10900, Thailand; 2 Kasetsart Vaccines and Bio-Product Innovation Centre, Kasetsart University, 50 Ngam Wong Wan, Chatuchak, Bangkok 10900, Thailand; 3 Coastal Fisheries Research and Development Bureau, Department of Fisheries, Ministry of Agriculture and Cooperatives, Bangkok 10900, Thailand; psoonson@hotmail.com (P.S.); pjumroensri@gmail.com (J.T.); varin_tana@hotmail.com (V.T.); 4 Department of Aquaculture, Faculty of Fisheries, Kasetsart University, 50 Ngam Wong Wan Road, Chatuchak, Bangkok 10900, Thailand; ffisnwa@ku.ac.th; 5 Department of Genetics, Faculty of Science, Kasetsart University, 50 Ngam Wong Wan, Chatuchak, Bangkok 10900, Thailand; fscitse@ku.ac.th

**Keywords:** comparative genome, metabolic pathway, pathogenesis, *Vibrio vulnificus*, virulence factors associated pathogenesis

## Abstract

Vibriosis caused by *Vibrio vulnificus* is a major problem in aquatic animals, particularly brown marble groupers (*Epinephelus fuscoguttatus*). *V. vulnificus* biotype I has recently been isolated and classified into subgroups SUKU_G1, SUKU_G2, and SUKU_G3 according to the different types of virulence genes. In a previous study, we have shown that biotype I *V. vulnificus* strains were classified into three subgroups according to the different types of virulence genes, which exhibited different phenotypes in terms of growth rate and virulence. To gain insight into the different genetic features revealed by the potential virulence mechanisms of *V. vulnificus* in relation to a spectrum of pathogenesis, comparative genomic analyses of three biotype I *V. vulnificus* strains belonging to different subgroups (SUKU_G1, SUKU_G2, and SUKU_G3) were performed. The *V. vulnificus* genome is composed of two circular chromosomes with average sizes of 3 Mbp and 1.7 Mbp that are evolutionarily related based on the analysis of orthologous genes. A comparative genome analysis of *V. vulnificus* revealed 5200 coding sequences, of which 3887 represented the core genome and the remaining 1313 constituted the dispensable genome. The most virulent isolate (SUKU_G1) carries unique enzymes that are important for lipopolysaccharide (LPS) and capsular polysaccharide (CPS) synthesis, as well as flagellar glycosylation, and harbors another type of repeat in toxin (RTX) and bacterial defense mechanisms. The less virulent isolate (SUKU_G2) shares enzymes related to CPS biosynthesis or flagellar glycosylation, while the avirulent isolate (SUKU_G3) and a less virulent isolate (SUKU_G2) share enzymes related to the production of rare sugars. Interestingly, the isolates from the three subgroups containing specific CMP-N-acetylneuraminate-producing enzymes that are correlated with their growth abilities. Collectively, these observations provide an understanding of the molecular mechanisms underlying disease pathogenesis and support the development of strategies for bacterial disease prevention and control.

## 1. Introduction

Vibriosis is a bacterial disease caused by *Vibrio* spp., such as *V. vulnificus*, *V. harveyi*, *V. ordalii*, *V. splendidus*, and *V. salmonicida*, that affects diverse marine fish and shellfish, leading to worldwide economic losses in the aquaculture industry [1,2]. Groupers are marine fish species with a mortality rate of approximately 66.7%, owing to vibriosis [3]. In Thailand, *V. vulnificus*, a Gram-negative halophilic bacterial pathogen [4], is a major bacterial species that causes diseases in the mariculture brown-marbled grouper (*Epinephelus fuscoguttatus*) [5].

In recent years, our laboratory isolated *V. vulnificus* biotype I bacteria from diseased brown-marbled groupers, which were classified into three subgroups (SUKU_G1, SUKU_G2, and SUKU_G3) based on the presence of virulence genes [6]. Importantly, all three STs show distinct pathogenicity, with the brown-marbled grouper, *V. vulnificus* SUKU_G1, exhibiting the highest virulence, whereas SUKU_G3 was non-virulent [6]. Moreover, the growth rate of *V. vulnificus* showed that SUKU_G1 and SUKU_G3 had the fastest growth rates compared to SUKU_G2 [6]. Sixteen common virulence genes were found in all the isolates, including *vvpE*, *gloB*, *tlh*, *Mann Hemo*, *ManIIA*, *rimO*, *vllY*, *ompU*, *vvhA*, *rtx*, *hlyIII*, *pilF*, *Mann TRAP*, *vcgC*, *16S rRNA type B*, and *nanA*, which are present in isolates classified as SUKU_G1. The SUKU_G2 isolates harbored four additional virulence genes (*YJ-like nab 1*, *YJ-like nab 2*, *cps*, and *sodA*), whereas SUKU_G3 isolates harbored three additional genes (*CM-like nab 1*, *CM-like nab2*, and *sodA*). Moreover, multilocus sequence typing (MLST) indicated that all isolated bacteria belonged to new sequence types (STs): SUKU_G1 (ST-595), SUKU_G2 (ST-596), and SUKU_G3 (ST-597).

Recently, whole-genome sequencing (WGS) has provided insights into antimicrobial resistance (AMR), metabolism, and the virulence of bacteria [7]. Pan-genomic reverse vaccinology compares the genomes of related bacterial strains to predict putative vaccine candidates and develop universal vaccines that are effective against different strains of the same pathogenic species [8,9], such as vaccines against *Escherichia coli* O157:H7 [10]. Moreover, synthetic biology and genome engineering can be used to develop novel diagnostic methods for infection detection, gain insight into antibiotic resistance mechanisms, and develop attenuated vaccine strains based on pathogenic bacterial genomes [7,11].

The genomes of *Vibrio* species typically have two chromosomes and mobile genetic elements, such as genomic islands (GI) and bacteriophages, that encode virulence and antibiotic resistance proteins [12]. Although all isolates of *V. vulnificus* were classified as biotype I, genomic information related to piscine pathogenesis is not available. Therefore, the genomes of the three subgroups, SUKU_G1, SUKU_G2, and SUKU_G3, were sequenced, and a comparative genome analysis was performed among the different strains to extend our understanding of the pathogenicity of *V. vulnificus* and related virulence factors at the genomic level.

## 2. Materials and Methods

### 2.1. Bacterial Strains

Three isolates of *V. vulnificus*, SUKU_G1, SUKU_G2, and SUKU_G3, were identified and characterized in Krabi, Thailand, between 2014 and 2017 [6]. Briefly, bacteria were isolated from the diseased groups and classified into three subgroups. The bacteria were cultured on Thiosulfate Citrate Bile Salt Sucrose agar to obtain single colonies. The sequence type of *V. vulnificus* in each group was verified using PCR testing of the virulence gene profile and an MLST analysis [6]. All isolated bacteria were stored at −80 °C until further use.

### 2.2. WGS and Assembly

SUKU_G1, SUKU_G2, and SUKU_G3 were cultured in tryptic soy broth (Difco, Franklin Lakes, NJ, USA) containing 1.5% sodium chloride at 30 °C, with shaking at 220 rpm for 16 h. Total genomic DNA was extracted using the DNeasy PowerSoil Pro Kit (QIAGEN, Hilden, Germany) according to the manufacturer’s instructions. The genome was sequenced using Illumina SBS technology in 2017 (Macrogen, Seoul, Republic of Korea) and converted into raw data for bioinformatic analysis. Raw sequence read data were tested for quality control using FastQC [13]. The reads were assembled using the reference genome of the pathogenic *V. vulnificus* strain CMCP6 biotype I (GenBank assembly accession: GCA_000039765) using the Burrows-Wheeler Aligner [14]. Variant information (single nucleotide polymorphisms (SNPs) and short indels) was obtained using SAMTools (http://www.htslib.org, accessed on 20 September 2017) through genomic alignment with the reference genome [15], and the aligned data were converted to FASTA format for further analysis. This whole-genome shotgun project was deposited in DDBJ/ENA/GenBank under accession numbers JAVKUT000000000 (SUKU_G1), JAVKUU000000000 (SUKU_G2), and JAVKUV000000000 (SUKU_G3). To construct the chromosome-level genome, the contigs were mapped and ordered into each chromosome of the reference genome using CONTIGuator (https://contiguator.sourceforge.net, accessed on 19 October 2021), a software tool for genome finishing, and the genome structure and contig profiling were visualized using the Artemis comparison tool [16,17].

### 2.3. Genome Annotation

The assembled genome was annotated with the Rapid Annotation Subsystem Technology (RAST) server v. 2.0 using the SEED framework (http://rast.nmpdr.org/, accessed on 25 October 2021) [18,19,20]. The virulence factor database (VFDB) was used to predict virulence factors based on BlastP search (http://www.mgc.ac.cn/VFs/main.htm, accessed on 30 October 2021) [21]. Genes potentially responsible for AMR in the *V. vulnificus* genome were identified using the Resistance Gene Identifier (RGI) with the Comprehensive Antibiotic Resistance Database (CARD) [22].

### 2.4. Mobile *Genetic Element Prediction*

Putative GI and prophages were predicted and compared using IslandCompare (https://islandcompare.ca/, accessed on 1 November 2021) [23] and PHAge Search Tool Enhanced Release (PHASTER) (https://phaster.ca/, accessed on 1 November 2021), respectively [24]. The *V. vulnificus* genome was searched for clustered regularly interspaced short palindromic repeats (CRISPR)/Cas system-associated locus using CRISPRminer (http://www.microbiome-bigdata.com/CRISPRminer/index.php/Home/Index/help, accessed on 3 November 2021) [25].

### 2.5. Comparative Whole-Genome Analysis

#### 2.5.1. Average Nucleotide Identity (ANI)

The average nucleotide identity values between the three piscine *V. vulnificus* genomes and the reference genome (CMCP6) were measured using the BLAST + (ANIb) program on the JSpeciesWS server (http://jspecies.ribohost.com/jspeciesws/, accessed on 2 December 2021) [26].

#### 2.5.2. Phylogenetic Tree Analysis

A phylogenetic tree was constructed from 3 piscine *V. vulnificus*, and 25 reference strains retrieved from the NCBI RefSeq database (Table S1). The core genome multilocus sequence typing (cgMLST) scheme was performed using SeqSphere+ (Ridom GmbH, Münster, Germany, http://www.ridom.de/seqsphere/index.shtml, accessed on 20 November 2024) with default parameters [27,28].

#### 2.5.3. Prediction of Clusters of Orthologous Groups (COGs)

Clusters of orthologous groups are clusters of proteins that are orthologous across at least three lineages and correspond to ancient, conserved domains. Clusters of orthologous groups were annotated and compared before the gene ontology (GO) enrichment analysis for the *V. vulnificus* genomes using the online program OrthoVenn2 (https://orthovenn2.bioinfotoolkits.net/start/db, accessed on 4 December 2021) with default parameters (E-value 1 × 10^−2^ and inflation value of 1.5) [29].

#### 2.5.4. Comparative Whole-Genome Visualization

Genome comparisons were performed among *V. vulnificus* strains using the BLAST Ring Image Generator (BRIG) [30]. A circular genomic map was drawn using the reference genome (*V. vulnificus* strain CMCP6) on a local BLAST + basis, with parameters 70% lower, a 90% upper cut-off for identity, and an E-value of 10. The ring color gradients correspond to varying degrees of the BLAST match identity. Circular genomic maps also included information on GC skew and content.

### 2.6. Pan-Core Genome Analysis

The bacterial pan-genome analysis tool was used to analyze the pan-core genomes of the three subgroups of *V. vulnificus* [31]. The program embedded MUSCLE and rsvg-convert for sequence alignment and tree generation [32]. The sGnuplot 4.6.6 program was used to plot the results. Functions and pathways were analyzed using BLASTKOALA based on the Kyoto Encyclopedia of Genes and Genomes ontology assignments [33].

## 3. Results

### 3.1. Genome Properties of Three Subgroups of V. vulnificus

The total base reads from the three subgroups of *V. vulnificus* showed more than 90% GC content similarity among the three isolates (chromosome I: 46.6–46.8% and chromosome II: 47–47.3%). The highest number of SNPs in the genome was observed in SUKU_G1, followed by SUKU_G2 and SUKU_G3, compared with the reference genome (*V. vulnificus* strain CMCP6). Nucleotide insertions and deletions were the lowest in the SUKU_G2 genome, with 275 deleted sites and 303 inserted sites, whereas SUKU_G1 and SUKU_G3 showed similar numbers of deleted and inserted sites. Mapped read data were assembled from the genome of *V. vulnificus* into two circular chromosomes, with GC content of approximately 46% in chromosome I and 47% in chromosome II. G1 had 3.2 Mbp in chromosome I (large) and 1.8 Mbp in chromosome II (small), whereas G2 and G3 contained 3 Mbp in the large chromosome and 1.7 Mbp in the small chromosome (Table 1).

**Figure 1:**
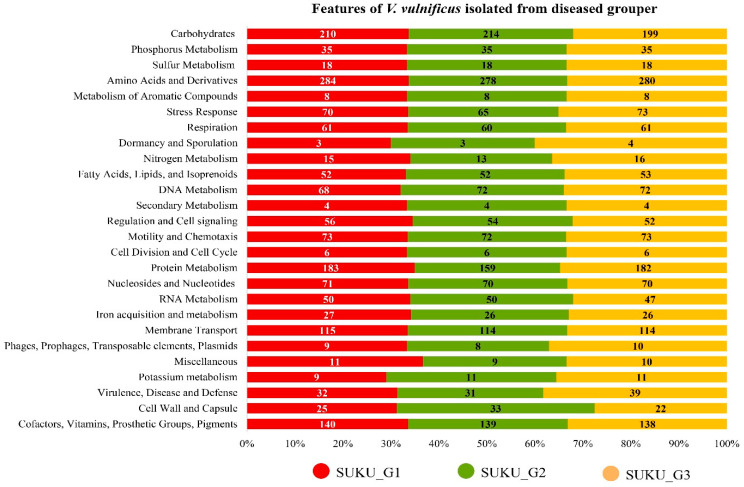
Functional annotation of predicted coding sequencing (CDs) from three strains of V. vulnificus (SUKU_G1, SUKU_G2, and SUKU_G3) isolated from diseased grouper using the SEED subsystem. Number represented the number of genes annotated within a particular function.

Genome annotation showed that G1 had the highest number (4670) of CDS, followed by 4510 in G2 and 4437 in G3. Approximately 30% of CDSs were functionally categorized into 367–371 subsystems. Among these subsystems, amino acids and derivatives, carbohydrates, and protein metabolism had the highest number of genes (Figure 1). SUKU_G2 had 20% fewer metabolism-related proteins than SUKU_G1 and SUKU_G3.

### 3.2. Identification of Genomic Islands (GI)

**Figure 2:**
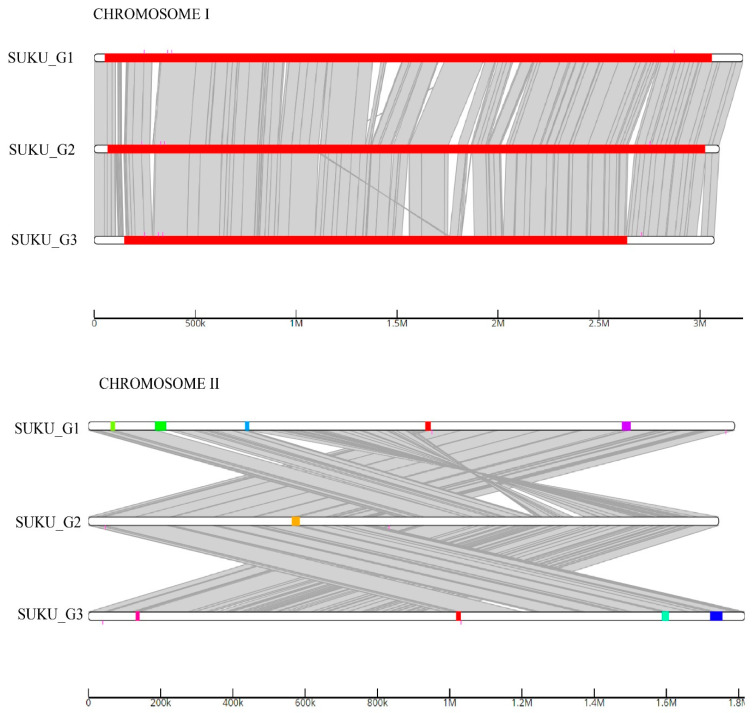
Comparative overview of genomic islands (GIs) found in the *V. vulnificus* genomes predicted using IslandCompare. Different box colors represent different clusters of GIs.

All isolates had one GI region in chromosome I, whereas chromosome II from SUKU_G1, SUKU_G2, and SUKU_G3 had four, five, and one GI regions, respectively (Figure 2). SUKU_G1 and SUKU_G2 shared GI regions that contained the 2-amino-3-ketobutyrate coenzyme A ligase and L-threonine 3-dehydrogenase genes.

### 3.3. Prophage Identification

Prophages in bacterial chromosomes play an important role in lateral gene transfer, harboring virulence factors and pathogenicity islands [34,35,36]. The PHASTER web server was used to annotate the putative prophage sequences in the *V. vulnificus* genome. In all three subgroups, the putative *Vibrio* prophage vB_VpaM_MAR was found in all three subgroups. *V. vulnificus* isolates SUKU_G1 and SUKU_G3 shared the *Klebsiella pneumoniae* ST147 VIM1phi7.1 phage, whereas SUKU_G2 and SUKU_G3 shared the *Bacillus* phage phBC6A51 (Table 2). SUKU_G1 had two unique prophages, *Enterobacteria* phage mEp235 and *Vibrio* phage PV94, whereas *Vibrio* phages 12B12 and KSF 1phi were found only in SUKU_G2, and *Vibrio* phages VfO3K6 and VFJ were found only in SUKU_G3. However, the majority of CDS of the predicted prophages encoded hypothetical proteins; therefore, it is unclear whether these prophages contribute to the virulence of this bacterium.

### 3.4. CRISPR Prediction

CRISPR has an immune function in bacteria and has been reported to be associated with virulence factors in some bacteria, such as *V. parahaemolyticus* [37]. To understand the role of CRISPR in the virulence and evolution of pathogens, CRISPR sequences and spacers were predicted. Only two CRISPR sequences were found in the SUKU_G1 genome, which is located on chromosomes I (2011365–2011478) and II (1251773–1251889), with one spacer at each site. The predicted genes in the 20-kb region and their detailed features are listed in Table S2.

### 3.5. Virulence Factors and AMR Profiles

Putative virulence factors involved in nutritional/metabolic pathways (heme biosynthesis and biotin synthesis), adherence (chitin-regulated pilus, mannose-sensitive HA, outer membrane protein U, Flp pili), type II and VI secretion system proteins, anti-phagocytosis (capsule), chemotaxis and motility, exotoxins, and exoenzymes (*RTX*, thermolabile hemolysin, leukocidin family pore-forming toxin (VvhA), and metallopeptidase) were observed in all three subgroups of *V. vulnificus* genomes (Table 3). Many putative virulence factors were observed in *V. cholerae*, *V. parahaemolyticus*, and other bacteria, including *Aeromonas* spp., *Acinetobacter* spp., *Haemophilus* spp., *Legionella* spp., *Pseudomonas* spp., and *E. coli*.

*V. vulnificus* strain SUKU_G1 has a unique pathway for the production of CMP-pseudaminic acid (CMP-Pse5Ac7Ac) (*Pse* gene) and LPS transport system (*wzx* and *wzy*), whereas the SUKU_G2 genome contains genes involved in the production of dTDP-4-amino-4,6-di-deoxy-D-galactose related to capsular polysaccharide biosynthesis. Different sets of genes involved in the capsular polysaccharide biosynthesis pathways, UDP-N-acetyl-D-mannosaminouronate (UDP-ManNAcA), UDP-N-acetyl-alpha-D-quinovosamine (UDP-QuiNAc), and UDP-N-acetyl-alpha-D-fucosamine (UDP-D-FucNAc), were observed in SUKU_G1 and SUKU_G2, whereas SUKU_G2 and SUKU_G3 contained genes involved in the CMP-N,N’-diacetyllegionaminate (CMP-leg5Ac7Ac) and UDP-N-acetyl-D-galactosaminuronic acid (UDP-GalNAcA) biosynthesis pathways (Table 4).

Based on RGI with CARD, 49 drug classes were included in the antibiotic resistance gene prediction program. With the default settings (perfect and strict), seven drug classes were found in these three subgroups, including cephamycin, cephalosporin, carbapenem, tetracycline, penam, fluoroquinolone, and macrolides (Table S3).

### 3.6. Phylogenetic Tree Analysis

**Figure 3:**
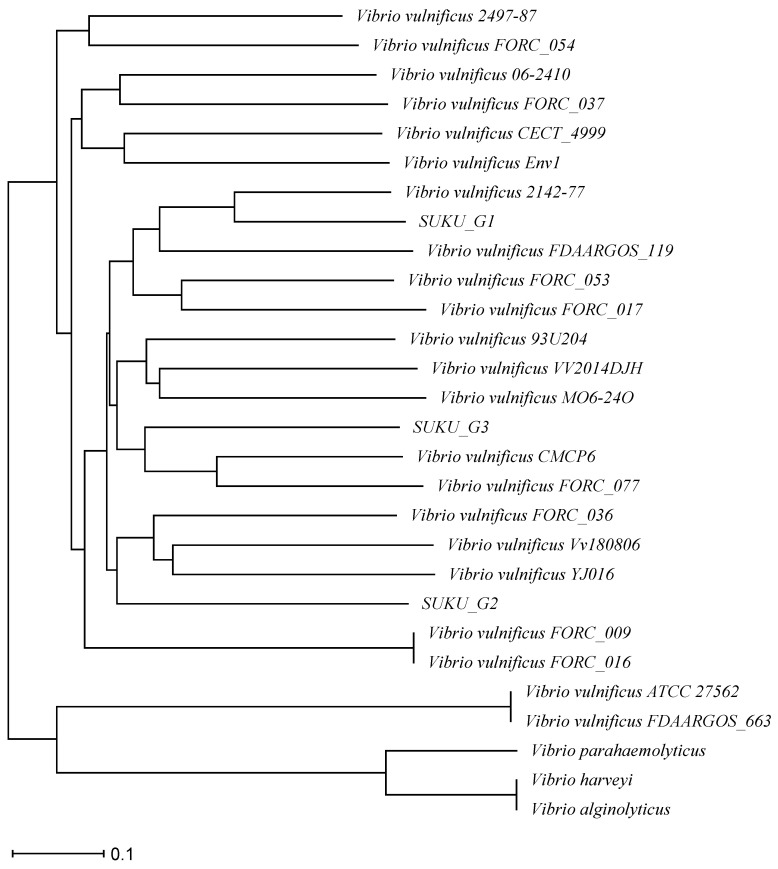
Phylogenetic tree constructed based on core genome multilocus sequence typing from *Vibrio* spp. (*V. vulnificus*, *V. alginolyticus*, *V. parahaemolyticus*, and *V. harveyi*).

Based on the ANI values, the close relationship among all piscine *V. vulnificus* samples in this study was supported by % ANI values higher than 98% (Table S4). SUKU_G3 was most closely related to the reference strain CMCP6. A phylogenetic tree was analyzed based on core genome multilocus sequence typing, which found that SUKU_G2 and SUKU_G3 belong to *V. vulnificus* strains YJ016 and CMCP6, respectively, while SUKU_G1 is related to *V. vulnificus* strain 2142-77. The outgroups, *V. harveyi*, *V. parahaemolyticus*, and *V. alginolyticus*, showed distinct distances from *V. vulnificus* (Figure 3).

**Figure 4:**
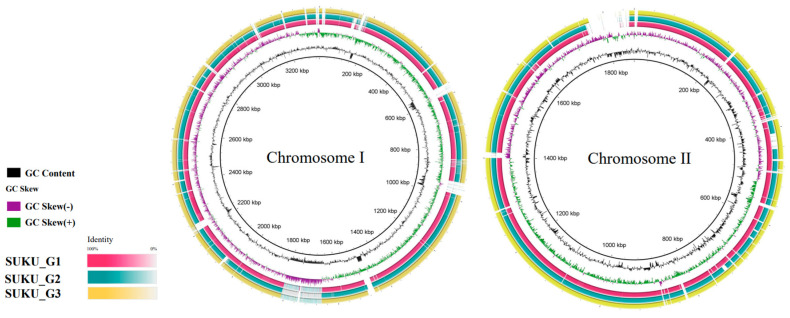
Comparative overview of *V. vulnificus* chromosomes of SUKU_G1 (pink), SUKU_G2 (green), and SUKU_G3 (yellow). Colored circles represent the genomes of each sequenced subgroup. The black circle represents the GC content, and the innermost circle represents the reference genome (CMCP6). The identity among the three subgroups is indicated by the color gradient.

To identify gene similarities and differences between the subgroups and CMCP6, a circular genomic map was constructed using BRIG, which revealed a high level of nucleotide identity among the three subgroups. Chromosomal architecture confirmed nucleotide conservation within certain regions of variation among the subgroups (Figure 4).

### 3.7. Orthologous Gene Analysis

An orthologous cluster gene analysis identified 3974 orthologous clusters shared among the three subgroups of *V. vulnificus*. SUKU_G1 and SUKU_G3 had the highest number of shared gene clusters (*n* = 100), followed by SUKU_G1 and SUKU_G2 (*n* = 95), indicating a close evolutionary relationship. SUKU_G1 contained the highest number of unique gene clusters (*n* = 11). One gene cluster was annotated as the histidine biosynthesis process (GO: 0000105). SUKU_G3 had the lowest number of unique gene clusters (*n* = 4), one of which was annotated as the DNA restriction-modification system GO:0009307 (Figure S1).

### 3.8. Pan-Core Genome Analysis

**Figure 5:**
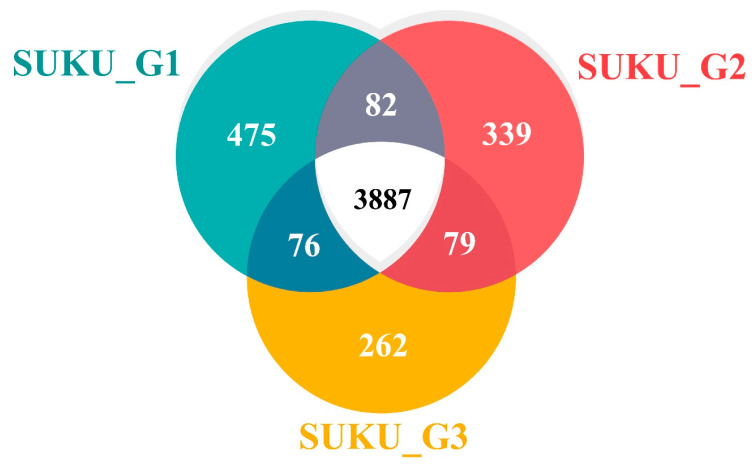
Venn diagram showing the core gene (white) and dispensable gene shared among the three subgroups (gray; SUKU_G1/SUKU_G2, dark red; SUKU_G2/SUKU_G3, and dark blue; SUKU_G1/SUKU_G3) and unique genes (green; SUKU_G1, red; SUKU_G2, and yellow; SUKU_G3) (right). Distribution of orthologous groups (functionally annotated or not) in different parts of the pan-genome based on the KEGG database (left).

**Figure 6:**
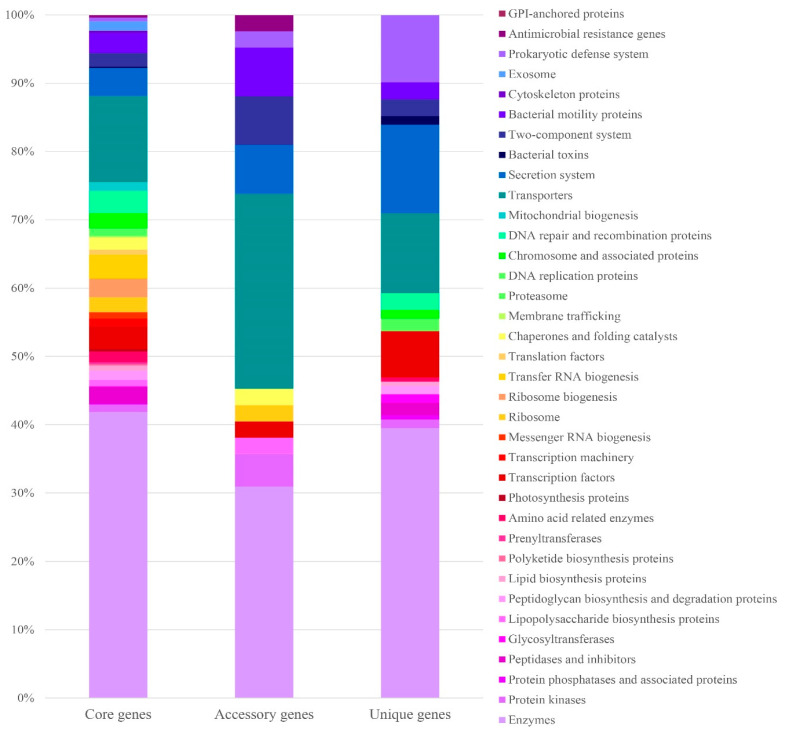
Distribution of functional classification of genes in the pan-genome of V. vulnificus samples. Colored bars represent the proportion of functional from core genes, accessory genes, and unique genes compared among three subgroups.

The pan-genome is the entire genome of a strain and is composed of a core genome and a dispensable genome. Core genes were present in all strains, whereas dispensable genes appeared only in a subset of strains. An analysis of the *V. vulnificus* pan-genome revealed 5200 CDS, of which 3887 were in the core genome, and the remaining 1313 constituted the dispensable genome, which corresponded to a circular genomic map representing the conservation and variation areas. (Figure 5). The dispensable genome was divided into 237 genes shared by the two strains and 1076 strain-specific genes. SUKU_G1 had the highest number of unique genes (475), whereas SUKU_G3 had the lowest (262). The core genome of *V. vulnificus* strains isolated from diseased groupers accounted for 74.75% of the average genome, indicating highly conserved genomic features. The majority of the core genes (64.6%) belonged to four main categories: metabolism, genetic information processing, signaling, and cellular processes, whereas accessory and unique genes were mainly categorized as unknown genes (Figure 6).

From the core genome, 1052 genes were categorized as metabolic enzymes from various pathways, such as carbohydrate, protein, lipid, and nucleotide metabolism, which normally function in energy supply; 615 genes were involved in genetic information processing, and the remaining 616 genes were involved in environmental information processing. Dispensable genes shared between strains were partially annotated as follows: 21.9% between SUKU_G1 and SUKU_G2 (82 genes), 13.1% between SUKU_G1 and SUKU_G3 (76 genes), and 22.7% between SUKU_G2 and SUKU_G3 (79 genes). These dispensable genes were involved in metabolic pathways, genetic information processing, transport, secretion systems, and prokaryotic defense systems. Specific genes from SUKU_G1 and SUKU_G2 were closely related, sharing a higher number of genes in the dispensable genome. Among the unique genes, 162 were functionally assigned, and 914 remained unknown. *V. vulnificus* strain SUKU_G1 had the highest number of unique genes, whereas SUKU_G3 had the lowest.

**Figure 7:**
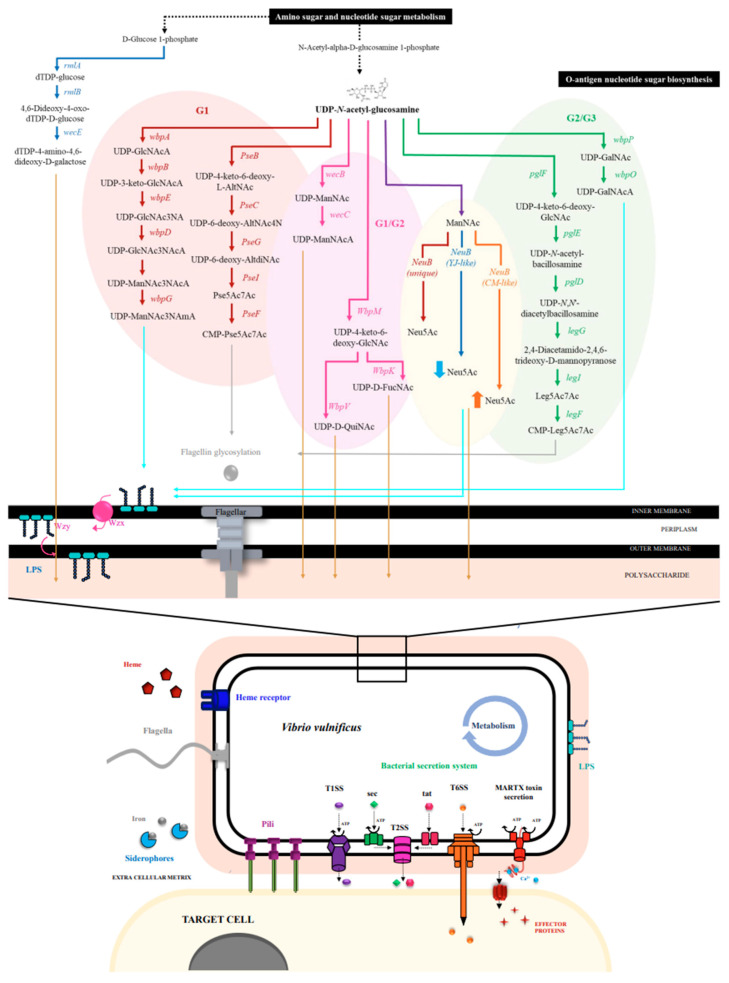
Purposed metabolic pathways associated with virulence and pathogenicity of *V. vulnificus* biotype I. The different arrow colors represent the presence of pathways found in different subgroups: red, G1; blue, G2; orange, G3; pink, G1/G2; and green, G2/G3.

The three subgroups of *V. vulnificus* had variable O-antigens that may be synthesized through different pathways that reflected their virulence. The *V. vulnificus* strain SUKU_G1 genome contained unique pathways for the synthesis of UDP-2,3-dideoxy-2-acetamido-3-acetamidino-mannuronic acid (UDP-ManNAc3NAmA) and CMP-Pse5Ac7Ac. UDP-ManNAc3NAmA is an unusual sugar and is synthesized by enzymes encoded by *wbpB*, *wbpE*, and *wbpD*; CMP- Pse5Ac7Ac is synthesized by enzymes encoded by *pseB, pseC*, *pseG, pseI*, and *pseF*. Subgroups SUKU_G1 and SUKU_G2 shared three pathways to produce UDP-ManNAcA, which is synthesized by enzymes encoded by *wecB* and *wecC*, and UDP-QuiNAc and UDP-D-FucNAc, which are synthesized by enzymes encoded by *wpM*, *wbpV*, and *wbpK,* respectively. The *V. vulnificus* strain SUKU_G2 and SUKU_G3 strains contained CMP-leg5Ac7Ac and UDP-GalNAcA, which are synthesized by enzymes encoded by *pglF*, *pglE*, *pglD*, *legG*, *legI*, *legF*, and *wbpP* and *wbpO*, respectively. Moreover, the gene that encodes the enzymes for the synthesis of CMP-N-acetylneuraminate (CMP-Neu5Ac) was different among the three subgroups; SUKU_G1 had a unique sequence, whereas SUKU_G2 and SUKU_G3 had genes similar to YJ-like nab and CM-like nab (Figure 7).

## 4. Discussion

Recently, many genomes of *V. vulnificus* biotype I have been isolated from humans, including strains YJ016, CMCP6, FORC_017, and MO6-24/O [38,39,40,41]. In fish, only *V. vulnificus* isolated from tilapia (strain 93U204) has been recorded [42], and none of the *V. vulnificus* genomes from groupers were present in any database. Thus, the WGSs of the three novel *V. vulnificus*, SUKU_G1, SUKU_G2, and SUKU_G3, isolated from groupers, exhibited different phenotypic and genotypic characteristics. All strains harbored two circular DNA chromosomes, which is consistent with the genomes of other *Vibrio* spp. [43]. The average nucleotide identity value is considered the best method for delineating bacterial species at the genome level and should be higher than 95% [44]. All grouper *V. vulnificus* and references shared an ANI value of over 98%, indicating that these strains belonged to the same species. The *V. vulnificus* strain SUKU_G3 had a higher ANI value and showed genetic relationships for strain CMCP6, which is in agreement with the presence of the CM-like *nab 1* and CM-like *nab2* alleles [6].

Antibiotic resistance, the CRISPR-Cas system, and virulence are important for bacterial survival; antibiotic resistance helps bacteria to deactivate drugs, CRISPR-Cas is an adaptive immune system, and virulence factors can establish pathogen infection. An antibiotic resistance analysis revealed that all subgroups carried antibiotic resistance genes to macrolides, fluoroquinolones, beta-lactams, and tetracycline with different resistance mechanisms, which related to our previous finding that SUKU_G1, SUKU_G2, and SUKU_G3 were multidrug-resistant strains [6]. However, *V. vulnificus* strains SUKU_G1 and SUKU_G3 were found to be sensitive to tetracycline (30 μg), whereas SUKU_G3 was susceptible to oxytetracycline (30 μg) [6]. The limitations of predicting resistance phenotypes from genotypic data may occur at the genetic level, as they are dependent on the mutations, copy number, and control of gene expression levels, which confer different susceptibility profiles [45,46].

Virulence factors are bacteria-associated molecules required for pathogenicity in host cell infection, including attachment, immunosuppression, and immune evasion [47]. The idea that “all subgroups carried antibiotic resistance genes to identification of virulence factors” is essential for understanding diseases, particularly when comparing virulent and avirulent subgroups. These bacteria can cause diseases through numerous virulence factors predicted by GI, VFDB, and pan-core genome analyses. An essential component of bacterial pathogens is the secretion of virulence factors in eukaryotic cells. Secretory proteins are translocated across the cytoplasmic membrane via either the general secretion pathway (Sec pathway) or twin-arginine translocation (Tat pathway), both of which are found in the core genome [48,49]. The pathogenic bacterium *V. parahaemolyticus* exports toxins through the Sec machinery to the periplasm, followed by secretion across the outer membrane via the type II secretion system (T2SS) [50]. Virulence factors from Gram-negative bacteria are transported outside the cell and, in some cases, directly into target cells via the secretion system. Secretion systems, including types I, II, and VI (T1SS, T2SS, and T6SS, respectively), are present in the core genome. Type I secretion systems export small molecules, such as antibiotics and toxins, out of the cell, as in *V. cholerae*, which uses its T1SS to secrete the MARTX toxin, thereby causing host cell rupture [51,52]. Type II secretion systems secrete a wide variety of folded exoproteins, including proteases, lipases, phospholipases, and toxins [53,54]. In *V. vulnificus*, T2SS is required for pathogenicity through the mediation of VvhA and VvpE secretion into the host cells [55]. Finally, type VI secretion systems are associated with bacterial toxin protein injection machinery for virulence in host cells [56].

Two major factors of virulence, capsular polysaccharide (CPS) and lipopolysaccharide (LPS), are differentially presented in different *V. vulnificus* subgroups. Capsular polysaccharides are components of the capsules that are used to avoid phagocytosis by host defense cells [57,58]. Lipopolysaccharide is an important pyrogen in *V. vulnificus* that causes endotoxic shock [59,60]. Both CPS and LPS are composed of flexible sugar polymers (O-antigens). Therefore, the types of sugars produced by each isolate may vary and may be related to their pathogenesis. The three subgroups of *V. vulnificus* have variable O-antigens, which may be synthesized through different pathways and reflect their virulence. The *V. vulnificus* strain SUKU_G1 genome contains unique pathways for the production of UDP-ManNAc3NAmA and CMP-Pse5Ac7Ac. An unusual sugar, UDP-ManNAc3NAmA, is found in the LPS of *Pseudomonas aeruginosa* PAO1 and is synthesized by *wbpB*-, *wbpE*-, and *wbpD*-encoded enzymes [61]. The CMP- Pse5Ac7Ac is sequentially transferred to flagellin monomers, and glycosylated flagellin monomers are secreted into the filament tip. Pseudaminic acids are present on the surface of pathogenic bacteria and are involved in flagellar glycosylation, which is necessary for the modification of flagellar assembly, bacterial motility, and colonization and, hence, the virulence of gastrointestinal pathogens [62,63,64].

The *V. vulnificus* SUKU_G1 and SUKU_G2 strains share three pathways for the production of UDP-ManNAcA, UDP-QuiNAc, and UDP-D-FucNAc. These sugars were discovered in CPS or LPS of various Gram-negative bacteria [62,65,66,67,68,69]. The *V. vulnificus* SUKU_G2 and SUKU_G3 strains had the CMP-leg5Ac7Ac and UDP-GalNAcA biosynthesis pathways, respectively. Legionaminic acids are critical *O*-linked modifications of Fla proteins and are essential for flagellar assembly, which is involved in the pathogenesis of Gram-negative bacteria [62]. UDP-GalNAcA is a component of the B-band O-antigen of *P. aeruginosa* [70].

Nucleotide sugar, CMP-Neu5Ac, is associated with lipooligosaccharides, which have been reported to contain terminal sialic acids in pathogenic bacteria, such as *Neisseria meningitidis* and *N. gonorrhoeae* [71,72]. The finding of unique genes correlated with our previous multiplex PCR results showed that the YJ-like nab allele demonstrated a low expression of NulOs in SUKU_G2, and the CM-like nab allele demonstrated a high expression of NulOs in SUKU_G3, as well as another sequence in SUKU_G1 [6]. Taken together, *V. vulnificus* strain SUKU_G1 has various sugar biosynthesis pathways, particularly the UDP-ManNAc3NAmA and CMP-Pse5Ac7Ac pathways, that are involved in pathogenesis.

Toxins are notable virulence factors of *Vibrio* spp. All *V. vulnificus* strains from groupers contained hemolysin, protease, and MARTX and its transport system, including the RtxBDE channel and TolC. Thermolabile hemolysin and hemolysin III proteins are putative pathogenic factors in many bacterial pathogens and are involved in bacterial bloodstream invasion [73]. Haemolysin may cause vasodilation, leading to hypotensive septic shock [74]. Exfoliative toxins A/B (chymotrypsin family of serine proteases) and collagenase can induce hemorrhagic damage and enhance vascular permeability and edema [75]. MARTX is a significant exotoxin that delivers various effector domains to host cells, ultimately inducing cell death [76]. It forms pores in the host cell membrane, facilitating the translocation and release of effector domains into the cells. The MARTX toxin identified in SUKU_G1 contains unique repeat domains and a galactose-binding-like domain, enabling binding to specific ligands on the host cell membrane [77,78,79,80]. This, coupled with the presence of RTX toxin determinant A and related Ca^2+^-binding proteins in SUKU_G1, as well as the presence of T6SS proteins, can contribute to its high virulence. Zonula occludens toxin, an enterotoxin that increases intestinal permeability, was discovered in SUKU_G2 and SUKU_G3, with different sequences that may function in pathogenesis.

Other minor virulence-related genes, such as Vibrio-associated phages, were observed among the three subgroups (vB_VpaM_MAR) and have also been observed in other *Vibrio* species in aquaculture. It should be noted that phage transfer may occur in aquatic animals [81]. The CRISPR-Cas system is a prokaryotic immune system that confers resistance to foreign genetic elements, such as those present within plasmids and phages, thereby providing a form of acquired immunity [82]. Only *V. vulnificus* strain SUKU_G1 contained CRISPR arrays that were identified as the orphan type, consisting only of palindromes and spacers without any *cas* genes. Quorum sensing is a cell-to-cell communication process that involves the production and release of extracellular compounds at high cell densities using autoinducers (AIs). The gene for AI-2 biosynthesis, *LuxS*, was found in our *V. vulnificus* strains and was present in all strains of *V. vulnificus*, regardless of biotype and serovar [83]. Outer membrane vesicles (OMVs) were produced by many pathogenic bacteria, including *V. vulnificus.* These vesicles are related to bacterial–host interactions, specifically their pathogenesis, as they modulate immune responses, contribute to biofilm formation, and deliver virulence factors to host cells, such as VvhA, which induces cytotoxicity [84,85,86]. Interestingly, the organization of *V. vulnificus* OMVs is affected by CPS expression [87].

Taken together, the *V. vulnificus* strain SUKU_G1 strain carried several genes related to virulence and pathogenesis, including those involved in the T6SS mechanism, the O-antigen nucleotide sugar biosynthesis pathway, and RTX toxin. The results of this study were consistent with our previous result that SUKU_G1 showed the fastest growth and highest pathogenicity (LD50 = 1.88 × 10^6^ CFU.mL^−1^). In contrast, the *V. vulnificus* SUKU_G3 strain had the same growth rate but no virulence effect on the fish. The *V. vulnificus* SUKU_G2 strain had less pathogenicity (LD50 = 7.63 × 10^6^ CFU.mL^−1^) than SUKU_G1 and had the slowest growth. Therefore, we proposed a pathogenic mechanism among these three subgroups of *V. vulnificus* biotype I, showing a correlation between the bacterial growth rate and pathogenicity.

A comparative genome analysis revealed that different virulence mechanisms among the three subgroups of *V. vulnificus* biotype I were correlated with pathogenicity. Understanding the virulence mechanisms has led to the development of prevention methods based on conserved virulence factors. The conserved features of virulent, less virulent, and avirulent strains are useful for vaccine design to prevent all types of this pathogen. Moreover, synthetic biology and genome engineering can be used to develop a novel diagnosis of infection using the different genes among the three subgroups, to understand antibiotic resistance mechanisms, and to develop attenuated vaccine strains from pathogenic bacterial genomes using the conserved phages found in this study.

## 5. Conclusions

This comparative genome analysis provides detailed insights into the diverse metabolic pathways of biotype I *V. vulnificus* strains. By combining the metabolic pathways with the pathogenicity of a strain, the virulence-related pathways could elucidate the potential virulence strategy of the bacteria. Hence, this alternated metabolic pathway, which both increases and decreases metabolites, as well as changes metabolism could be affected by different environments or specific triggers that co-opt the virulence of bacteria. This system is known as bacterial virulence programming, which results in the enhancement of bacterial virulence, including changes in bacterial characteristics or properties, such as from acute to chronic infection, from local to systemic infection, and from infection to colonization [88]. Taken together, this is the first report of the association between bacterial pathogenicity and virulent pathways in piscine *V. vulnificus* that not only provides an understanding of relevant metabolic and virulence pathways but also supports the design of disease prevention and control methods. The results of this study provide insights into the genetic differences among the three subgroups. The main virulence factors associated with pathogenesis may be useful for controlling this virulent pathogen in brown-marbled groupers.

## Figures and Tables

**Table 1:** Summary of assembly and annotation characteristics for *V. vulnificus*.

	SUKU-G1		SUKU-G2		SUKU-G3	
Chromosome	I	II	I	II	I	II
Total read (Mbp)	8.8		6.2		9.5	
Reference length (Mbp)	5.1					
Mapped sites (Mbp)	4.7 (91.75%)		4.6 (90.41%)		4.6 (91.5%)	
Number of contigs	207		209		66	
SNPs	67,907		66,806		63,273	
Insertion/deletion	300/340		275/303		306/344	
Chromosomes number	2		2		2	
Mapped contigs	66	31	68	31	40	1
Mapped length (Mbp)	3.2	1.8	3.0	1.7	3.0	1.7
GC content (%)	46.6	47	46.8	47.2	46.7	47.3
Number of coding sequences	3041	1629	2872	1638	2901	1536
Number of subsystems	371		368		367	
Number of RNA						
rRNA	1		-		-	
tRNA	48		37		62	

**Table 2:** Prophage identification from *V. vulnificus* genomes.

Predicted Prophage	SUKU_G1	SUKU_G2	SUKU_G3
Phage *Vibrio* vB VpaM MAR	+	+	+
Phage *Klebsi* ST147 VIM1phi7.1	+	−	+
Phage *Entero* mEp235	+	−	−
Phage *Vibrio* PV94	+	−	−
Phage *Bacill* phBC6A51	−	+	+
Phage *Vibrio* 12B12	−	+	−
Phage *Vibrio* KSF 1phi	−	+	−
Phage *Vibrio* VfO3K6	−	−	+
Phage *Vibrio* VFJ	−	−	+

+, present; −, absent.

**Table 3:** Potential virulence factor profiles of the three *V. vulnificus* strains predicted by the virulence factor database.

Classification	Virulence Factors	Related Genes	Chr.
Nutritional/ Metabolic factor	Heme biosynthesis (*Haemophilus*)	*hemA*, *hemB*, *hemC*, *hemD*, *hemE*, *hemG*, *hemH*, *hemL*, *hemM*, *hemN*, *hemX*, *hemY*	I
	CcmC (*Legionella*)	*ccmA*, *ccmB*, *ccmC*, *ccmE*, *ccmF*	I
	Biotin synthesis (*Francisella*)	*bioA*, *bioB*, *bioD*, *bioF*	I
	Vibriobactin/ Vulnibactin	*vibA*, *vibB*, *vibC*, *vibE*, *vibF*, *vibH*, *viuA*, *viuB*, *BJE04_RS21740*, *BJE04_RS21750*, *BJE04_RS21780*, *BJE04_RS21785*, *BJE04_RS21790*	II
	VctPDGC system	*vctP*, *vctD*, *vctG*, *vctC*	II
	Aerobactin (*Escherichia*)	*iutA*	II
	Heme receptor	*hutA*, *hutR*	II
	HuvA (*Listonella*)	*huvB*, *huvC*, *huvD*, *huvX*, *hucZ*	II
Adherence	ChiRP	*pilA*, *pilB*, *pilC*, *pilD/vcpD*, *pilM*, *pilN*, *pilO*, *pilP*, *pilQ*, *pilT*, *PilT/PilU*, *VV1_RS01710*, *VV1_RS01705*, *VV1_RS01700*,	I
	Mannose-sensitive HA	*mshA-N*	I
	Immunogenic lipoprotein A	*IlpA*	I
	Outer membrane protein U	*ompU*	I
	Flp type IV pili (*Aeromonas*)	*flpA-L*, *flp1*	I
	Accessory colonization factor	*acfB*, *acfD*	I
	Heat shock protein 60	*hspB*	I,II
	N-acetylglucosamine-binding protein	*gbpA*	II
	Flp pili	*VV1_RS15585*, *VV1_RS15590*, *VV1_RS15595*, *VV1_RS15600*, *VV1_RS15605*, *VV1_RS15610*, *VV1_RS15615*, *VV1_RS15620*, *VV1_RS15625*, *VV1_RS15630*, *VV1_RS15635*, *VV1_RS15640*	II
Effector delivery system	Eps T2SS	*epsC-N*, *VV1_RS03010*	I
	T6SS HSI-I (*Pseudomonas*)	*vgrG1a*, *hcp1*, *clpV1*, *hsiH1/tssG*, *hsiG1/tssF*, *hsiF1/tssE*, *hsiE1*, *hsiC1/vipB/tssC*, *hsiB1/vipA/tssB*, *hsiA1*, *pppA*, *tagF/pppB*, *icmF1/tssM1*, *dotU1*, *hsiJ1*, *lip1*, *fha1*, *ppkA*	II
Immune modulation	LOS (*Haemophilus*)	*htrB*, *waaQ*, *lgtF*, *kdkA*, *msbA*, *msbB*, *kpsF*, *kdsA*, *gmhA/lpcA*, *opsX/rfaC*, *rfaD*, *rfaE*, *lpxA-E*, *lpxH*, *lpxK*, *orfM*,	I
	LOS	*manB/yhxB*	II
	LPS (*Pseudomonas*)	*hisH2*, *hisF2*, *waaA*, *waaF*	I
	Capsule (*Klebsiella*)	*gndA*	I
	Capsule (*Francisella*)	*rpe*	I
	Capsular polysaccharide	*wbfB*, *wbfC*, *wbpM*, *ugd*, *wza-c*	I
		*cpsA-D*, *cpsF*, *cpsH-J*	II
	Exopolysaccharide (*Haemophilus*)	*mrsA/glmM*, *pgi*	I
		*manA*	II
Chemotaxis and motility	Flagella	*cheA*, *cheB*, *cheR*, *cheV*, *cheW*, *cheY*, *cheZ*, *flgA-O*, *flgT*, *flaA-E*, *flaG*, *flaI*, *flhA*, *flhB*, *flhF*, *fleN/flhG*, *flrA*, *fleS/flrB*, *fleR/flrC*, *fliA*, *fliD-S*, *motA*, *motB*, *motX*, *motZ*	I
Regulation	Two-component system (*Acinetobacter*)	*bfmR*, *bfmS*	I
	GacS/GacA two-component system (*Pseudomonas*)	*GacS*, *GacA*	I
Biofilm	Alginate (*Pseudomonas*)	*mucA-D*, *mucP*, *algB*, *algU*, *algR*, *algW*, *algZ*	I
	VPS	*vpsE*, *vpsG*, *vpsH*, *vpsI*, *vpsU*	I
	AI-2	*luxS*	I
Stress survival	catalase-peroxidase (*Legionella*)	*katB*	I
		*katA*	II
	superoxide dismutase (*Legionella*)	*sodB*	I
		*sodCI*	II
	Clp protease proteolytic (*Listeria*)	*clpP*	I
Exotoxin	Thermolabile hemolysin	*tlh*	II
	Leukocidin family pore-forming toxin	*vvhA*	II
	Repeat in toxin	*rtxA-E*, *rtxH*	II
Exoenzyme	Metallopeptidase	*hap/vvp*	II

CcmC, cytochrome c maturation; ChiRP, chitin-regulated pilus; T6SS, type 6 secretion system; LOS, lipooligosaccharides; LPS, lipopolysaccharides; VPS, Vibrio polysaccharide; AI-2, cholerae autoinducer-2; Eps, extracellular protein secretion.

**Table 4:** Different potential virulence genes among three subgroups of *V. vulnificus*.

Virulence Factor	Gene Function	Gene	G1	G2	G3
LPS	DegT/DnrJ/EryC1/StrS family aminotransferase	*wecE*	/	/	
	acetyltransferase	*pglD*		/	/
	O-antigen translocase	*wzx*	/		
	B-band O-antigen polymerase	*wzy*	/		
	Vi polysaccharide biosynthesis UDP-N-acetylglucosamine C-6 dehydrogenase TviB	*tviB*			/
Capsule	UDP-N-acetylglucosamine 4,6-dehydratase	*PseB*	/		
	UDP-4-amino-4, 6-dideoxy-N-acetyl-beta-L-altrosamine transaminase	*PseC*	/		
	pseudaminic acid biosynthesis PseA protein	*PseA*	/		
	pseudaminic acid cytidylyltransferase	*PseF*	/		
	UDP-2,4-diacetamido-2,4, 6-trideoxy-beta-L-altropyranose hydrolase	*PseG*	/		
	pseudaminic acid synthase	*PseI*	/		
	Vi polysaccharide biosynthesis UDP-N-acetylglucosamine C-6 dehydrogenase TviB	*tviB*	/		
	MviM protein	*mviM*	/		
	WbbJ protein	*wbbJ*	/		
	WecE protein	*wecE*	/		
	UDP-N-acetylglucosamine 4,6-dehydratase	*pglF*		/	/
	LegC family aminotransferase	*pglE*		/	/
	N,N’-diacetyllegionaminate synthase (O-linked)	*legl*		/	/
	UDP-N-acetylglucosamine 2-epimerase	*legG*		/	/
	acylneuraminate cytidylyltransferase family	*legF*		/	/
	Vi polysaccharide biosynthesis UDP-N-acetylglucosaminuronic acid C-4 epimerase TviC	*tviC*	/		/
Capsular polysaccharide	dTDP-glucose 4,6-dehydratase	*rmlB*		/	
	glucose-1-phosphate thymidylyltransferase	*rmlA*		/	
	NAD-dependent epimerase/dehydratase family protein	*wbpK*	/	/	
	sugar transferase	*wbfU*	/	/	
	UDP-N-acetylglucosamine 2-epimerase (non-hydrolyzing)	*wbjD/wecB*	/	/	
	UDP-N-acetyl-D-mannosamine dehydrogenase	*wecC*	/	/	
	sugar transferase	*wcaJ*			/

## Data Availability

The original contributions presented in the study are included in the article/Supplementary Material, further inquiries can be directed to the corresponding authors.

## Supplementary Materials

The following supporting information can be downloaded at https://www.mdpi.com/article/10.3390/microorganisms12122518/s1: Figure S1: Venn diagram showed the distribution of shared and unique orthologous gene clusters among *V. vulnificus* samples by OrthoVenn2. Figure S2: Subsystem category distribution based on RAST annotation server. Table S1: Reference genomes of *V. vulnificus* and other *Vibrio* species. Table S2: The predicted genes in the 20-kb regions and detailed features from CRISPR prediction. Table S3: Antibiotic resistance profile predicted from CARD. Table S4: ANI value between piscine *V. vulnificus* and strain CMCP6. The asterisk (*) represents the ANI value compared to itself.

## References

[1] Chatterjee S., Haldar S. (2012). Vibrio related diseases in aquaculture and development of rapid and accurate identification methods. J. Mar. Sci. Res. Dev..

[2] Toranzo A.E., Magarin B., Romalde J.L. (2005). A review of the main bacterial fish diseases in mariculture systems. Aquaculture.

[3] Chong R., Bousfield B., Brown R. (2011). Fish disease management. Vet. Bull. Agric. Fish. Conserv. Dep. Newsl..

[4] Tacket C., Brenner F., Blake P.A. (1984). Clinical features and epidemiological study of *Vibrio vulnificus* infections. J. Infect. Dis..

[5] Thawonsuwan J., Kasornchandra J., Soonsan P., Keawtapee C. (2016). Isolation of *Vibrio vulnificus* biotype I from disease outbreaks on cultured tiger grouper *Epinephelus fuscoguttatus* Forsskal, 1775. Fish Pathol..

[6] Hoihuan A., Soonson P., Bunlipatanon P., Thawonsuwan J., Tanasomwang V., Areechon N., Unajak S. (2021). Molecular genotyping and phenotyping of *Vibrio vulnificus* isolated from diseased, brown-marbled grouper (*Epinephelus fuscoguttatus*) in Thailand with preliminary vaccine efficacy analysis. Aquaculture.

[7] Talagrand-Reboul E., Colston S.M., Graf J., Lamy B., Jumas-Bilak E. (2020). Comparative and evolutionary genomics of isolates provide insight into the pathoadaptation of *Aeromonas*. Genome Biol. Evol..

[8] Vernikos G. (2008). Overtake in reverse gear. Nat. Rev. Microbiol..

[9] Mora M., Donati C., Medini D., Covacci A., Rappuoli R. (2006). Microbial genomes and vaccine design: Refinements to the classical reverse vaccinology approach. Curr. Opin. Microbiol..

[10] García-Angulo V.A., Kalita A., Kalita M., Lozano L., Torres A.G. (2014). Comparative genomics and immunoinformatics approach for the identification of vaccine candidates for enterohemorrhagic *Escherichia coli* O157:H7. Infect. Immun..

[11] Krishnamurthy M., Moore R.T., Rajamani S., Panchal R.G. (2016). Bacterial genome engineering and synthetic biology: Combating pathogens. BMC Microbiol..

[12] Sobecky P.A., Hazen T.H. (2009). Horizontal gene transfer and mobile genetic elements in marine systems. Methods Mol. Biol..

[13] FASTQC (2010). A Quality Control Tool for High Throughput Sequence Data. http://www.bioinformatics.babraham.ac.uk/projects/fastqc/.

[14] Li H., Durbin R. (2009). Fast and accurate short read alignment with Burrows-Wheeler Transform. Bioinformatics.

[15] Li H., Handsaker B., Wysoker A., Fennell T., Ruan J., Homer N., Marth G., Abecasis G., Durbin R. (2009). The sequence alignment/map format and SAMtools. Bioinformatics.

[16] Galardini M., Biondi E.G., Bazzicalupo M., Mengoni A. (2011). CONTIGuator: A bacterial genome finishing tool for structural insights on draft genomes. Source Code Biol. Med..

[17] Carver T., Berriman M., Tivey A., Patel C., Böhme U., Barrell B.G., Parkhill J., Rajandream M.A. (2008). Artemis and ACT: Viewing; annotating, and comparing sequences stored in a relational database. Bioinformatics.

[18] Aziz R.K., Bartels D., Best A.A., DeJongh M., Disz T., Edwards R.A., Formsma K., Gerdes S., Glass E.M., Kubal M. (2008). The RAST Server: Rapid annotations using subsystems technology. BMC Genom..

[19] Overbeek R., Olson R., Pusch G.D., Olsen G.J., Davis J.J., Disz T., Edwards R.A., Gerdes S., Parrello B., Shukla M. (2014). The SEED and the rapid Annotation of microbial genomes using subsystems technology (RAST). Nucleic Acids Res..

[20] Brettin T., Davis J.J., Disz T., Edwards R.A., Gerdes S., Olsen G.J., Olson R., Overbeek R., Parrello B., Pusch G.D. (2015). RASTtk: A modular and extensible implementation of the RAST algorithm for building custom annotation pipelines and annotating batches of genomes. Sci. Rep..

[21] Liu B., Zheng D., Jin Q., Chen L., Yang J. (2019). VFDB 2019: A comparative pathogenomic platform with an interactive web interface. Nucleic Acids Res..

[22] Alcock B.P., Raphenya A.R., Lau T.T.Y., Tsang K.K., Bouchard M., Edalatmand A., Huynh W., Nguyen A.L.V., Cheng A.A., Liu S. (2020). CARD 2020: Antibiotic resistome surveillance with the comprehensive antibiotic resistance database. Nucleic Acids Res..

[23] Bertelli C., Gray K.L., Woods N., Lim A.C., Tilley K.E., Winsor G.L., Hoad G.R., Roudgar A., Spencer A., Peltier J. (2022). Enabling genomic island prediction and comparison in multiple genomes to investigate bacterial evolution and outbreaks. Microb. Genom..

[24] Arndt D., Grant J.R., Marcu A., Sajed T., Pon A., Liang Y., Wishart D.S. (2016). PHASTER: A better, faster version of the PHAST phage search tool. Nucleic Acids. Res..

[25] Zhang F., Zhao S., Ren C., Zhu Y., Zhou H., Lai Y., Zhou F., Jia Y., Zheng K., Huang Z. (2018). CRISPRminer is a knowledge base for exploring CRISPR-Cas systems in microbe and phage interactions. Commun. Biol..

[26] Richer M., Rosselló-Móra R., Glöckner F.O., Peplies J. (2016). JSpeciesWS: A web server for prokaryotic species circumscription based on pairwise genome comparison. Bioinformatics.

[27] Ghanem M., El-Gazzar M. (2019). Development of a multilocus sequence typing assay for *Mycoplasma gallisepticum*. Avian Dis..

[28] Menghwar H., Guo A., Chen Y., Lysnyansky I., Parker A.M., Prysliak T., Perez-Casal J. (2022). A core genome multilocus sequence typing (cgMLST) analysis of *Mycoplasma bovis* isolates. Vet. Microbiol..

[29] Xu L., Dong Z., Fang L., Luo Y., Wei Z., Guo H., Zhang G., Gu Y.Q., Coleman-Derr D., Xia Q. (2019). OrthoVenn2: A web server for whole-genome comparison and annotation of orthologous clusters across multiple species. Nucleic Acids. Res..

[30] Alikhan N.F., Petty N.K., Zakour N.L.B., Beatson S.A. (2011). BLAST Ring Image Generator (BRIG): Simple prokaryote genome comparisons. BMC Genom..

[31] Chaudhari N., Gupta V., Dutta C. (2016). BPGA- an ultra-fast pan-genome analysis pipeline. Sci. Rep..

[32] Edgar R.C. (2004). MUSCLE: Multiple sequence alignment with high accuracy and high throughput. Nucleic Acids Res..

[33] Kanehisa M., Sato Y., Morishima K. (2016). BlastKOALA and GhostKOALA: KEGG tools for functional characterization of genome and metagenome sequences. J. Mol. Biol..

[34] Canchaya C., Fouurnous G., Chibani-Chennoufi S., Dillmann M.-L., Brussow H. (2003). Phage as agents of lateral gene transfer. Curr. Opin. Microbiol..

[35] Wagner P.L., Waldor M.K. (2002). Bacteriophage control of bacterial virulence. Infect. Immun..

[36] Boyd E.F., Davis B.M., Hochhut B. (2001). Bacteriophage-bacteriophage interactions in the evolution of pathogenic bacteria. Trends Microbiol..

[37] Sun H., Li Y., Shi X., Lin Y., Qiu Y., Zhang J., Liu Y., Jiang M., Zhang Z., Chen Q. (2015). Association of CRISPR/Cas evolution with *Vibrio parahaemolyticus* virulence factors and genotypes. Foodborne Pathog. Dis..

[38] Chen C.Y., Wu K.-M., Chang Y.-C., Chang C.-H., Tsai H.-C., Liao T.-L., Liu Y.-M., Chen H.-J., Shen A.B.-T., Li J.-C. (2003). Comparative genome analysis of *Vibrio vulnificus*, a marine pathogen. Genome Res..

[39] Kim Y.R., Lee S.E., Kim C.M., Kim S.Y., Shin E.K., Shin D.H., Chung S.S., Choy H.E., Progulske-Fox A., Hillman J.D. (2003). Characterization and pathogenic significance of *Vibrio vulnificus* antigens preferentially expressed in septicemic patients. Infect. Immun..

[40] Park J.H., Cho Y.-J., Chun J., Seok Y.-J., Lee J.K., Kim K.-S., Lee K.-H., Park S.-J., Choi S.H. (2011). Complete genome sequence of *Vibrio vulnificus* MO6-24/O. J. Bacteriol..

[41] Chung H.Y., Kim Y.-T., Kim S., Na E.J., Ku H.-J., Lee K.H., Heo S.T., Ryu S., Kim H., Choi S.H. (2016). Complete genome sequence of *Vibrio vulnificus* FORC_017 isolated from a patient with a hemorrhagic rash after consuming raw dotted gizzard shad. Gut Pathog..

[42] Lo W.S., Chen H., Chen C.-Y., Kuo C.-H. (2014). Complete genome sequence of *Vibrio vulnificus* 93U204, a bacterium isolated from diseased tilapia in Taiwan. Genome Announc..

[43] Okada K., Iida T., Kita-Tsukamoto K., Honda T. (2005). Vibrios commonly possess two chromosomes. J. Bacteriol..

[44] Richter M., Rosselló-Móra R. (2009). Shifting the genomic gold standard for the prokaryotic species definition. Proc. Natl. Acad. Sci. USA.

[45] Depardieu F. (2007). Modes and modulations of antibiotic resistance gene expression. Clin. Microbiol. Rev..

[46] Nicoloff H. (2019). The high prevalence of antibiotic heteroresistance in pathogenic bacteria is mainly caused by gene amplification. Nat. Microbiol..

[47] Sharma A.K., Dhasmana N., Dubey N., Kumar N., Gangwal A., Gupta M., Singh Y. (2017). Bacterial virulence factors: Secreted for survival. Indian J. Microbiol..

[48] Driessen A.J.M., Manting E.H., Does C.V.D. (2001). The structural basis of protein targeting and translocation in bacteria. Nat. Struct. Biol..

[49] Berks B.C., Palmer T., Sargent F. (2005). Protein targeting by the bacterial twin-arginine translocation (Tat) pathway. Curr. Opin. Microbiol..

[50] Matsuda S., Okada R., Tandhavanant S., Hiyoshi H., Gotoh K., Iida T., Kodama T. (2019). Export of a *Vibrio parahaemolyticus* toxin by the Sec and type III secretion machineries in tandem. Nat. Microbiol..

[51] Dolores J.S., Agarwal S., Egerer M., Satchell K.J.F. (2015). *Vibrio cholerae* MARTX toxin heterologous translocation of beta-lactamase and roles of individual effector domains on cytoskeleton dynamics. Mol. Microbiol..

[52] Symmons M.F., Bokma E., Koronakis E., Hughes C., Koronakis V. (2009). The assembled structure of a complete tripartite bacterial multidrug efflux pump. Proc. Natl. Acad. Sci. USA.

[53] Korotkov K.V., Sandkvist M., Hol W. (2012). The type II secretion system: Biogenesis, molecular architecture and mechanism. Nat. Rev. Microbiol..

[54] Korotkov K.V., Sandkvist M. (2019). Architecture, function, and substrates of the type II secretion system. EcoSal Plus.

[55] Hwang W., Lee N.Y., Kim J., Lee M.A., Kim K.-S., Lee K.-H., Park S.-J. (2011). Functional characterization of EpsC, a component of the type II secretion system, in the pathogenicity of *Vibrio vulnificus*. Infect. Immun..

[56] Cherrak Y., Flaugnatti N., Durand E., Journet L., Cascales E. (2019). Structure and activity of the type VI secretion system. Microbiol. Spectrum..

[57] Hayat U., Reddy G.P., Bush C.A., Johnson J.A., Wright A.C., Morris J.G. (1993). Capsular types of *Vibrio vulnificus*: An analysis of strains from clinical and environmental sources. J. Infect. Dis..

[58] Simonson J.G., Siebeling R.J. (1993). Immunogenicity of *Vibrio vulnificus* capsular polysaccharides and polysaccharide-protein conjugates. Infect. Immun..

[59] McPherson V.L., Watts J.A., Simpson L.M., Oliver J.D. (1991). Physiological effects of the lipopolysaccharide of *Vibrio vulnificus* on mice and rats. Microbios.

[60] Jones M.K., Oliver J.D. (2009). *Vibrio vulnificus*: Disease and pathogenesis. Infect. Immun..

[61] Westman E.L., McNally D.J., Charchoglyan A., Brewer D., Field R.A., Lam J.S. (2009). Characterization of WbpB, WbpE, and WbpD and reconstitution of a pathway for the biosynthesis of UDP-2,3-diacetamido-2,3-dideoxy-D-mannuronic acid in *Pseudomonas aeruginosa*. J. Biol. Chem..

[62] Knirel Y.A., Vinogradov E.V., L’vov V.L., Kocharova N.A., Shashkov A.S., Dmitriev B.A., Kochetkov N.K. (1984). Sialic acids of a new type from the lipopolysaccharides of *Pseudomonas aeruginosa* and *Shigella boydii*. Carbohydr. Res..

[63] Schirm M., Soo E.C., Aubry A.J., Austin J., Thibault P., Logan S.M. (2003). Structural, genetic and functional characterization of the flagellin glycosylation process in *Helicobacter pylori*. Mol. Microbiol..

[64] Thibault P., Logan S.M., Kelly J.F., Brisson J.-R., Ewing C.P., Trust T.J., Guerry P. (2001). Identification of the carbohydrate moieties and glycosylation motifs in *Campylobacter jejuni* flagellin. J. Biol. Chem..

[65] Mayer H. (1969). D-Mannosaminuronsäure-Baustein des K7 antigens von *Escherichia coli*. Eur. J. Biochem..

[66] Torii M., Sakakibara K., Kuroda K. (1973). Occurrence of 2-amino-2-deoxy-hexuronic acids as constituents of *Vibrio parahaemolyticus* K15 antigen. Eur. J. Biochem..

[67] Cava J.R., Elias P.M., Turowski D.A., Noel K.D. (1989). *Rhizobium leguminosarum* CFN42 genetic regions encoding lipopolysaccharide structures essential for complete nodule development on bean plants. J. Bacteriol..

[68] Bélanger M., Burrows L.L., Lam J.S. (1999). Functional analysis of genes responsible for the synthesis of the B-band O-antigen of *Pseudomonas aeruginosa* serotype O6 lipopolysaccharide. Microbiology.

[69] Forsberg L.S., Noel K.D., Box J., Carlson R.W. (2003). Genetic locus and structural characterization of the biochemical defect in the O-antigenic polysaccharide of the symbiotically deficient *Rhizobium etli* mutant, CE166. Replacement of N-acetylquinovosamine with its hexosyl-4-ulose precursor. J. Biol. Chem..

[70] Mulrooney E.F., Poon K.K.H., McNally D.J., Brisson J.-R., Lam J.S. (2005). Biosynthesis of UDP-N-acetyl-L-fucosamine, a precursor to the biosynthesis of lipopolysaccharide in *Pseudomonas aeruginosa* Serotype O11*. J. Biol. Chem..

[71] Campagnari A.A., Spinola S.M., Lesse A.J., Kwaik Y.A., Mandrell R.E., Apicello M.A. (1990). Lipooligosaccharide epitopes shared among Gram negative non-enteric mucosal pathogens. Microb. Pathog..

[72] Mandrell R.E., Apicella M.A. (1993). Lipo-oligosaccharides (LOS) of mucosal pathogens: Molecular mimicry and host-modification of LOS. Immunobiology.

[73] Senoh M., Miyoshi S.-I., Okamoto K., Fouz B., Amaro C., Shinoda S. (2005). The cytotoxin-hemolysin genes of human and eel pathogenic *Vibrio vulnificus* strains: Comparison of nucleotide sequences and application to genetic grouping. Microbiol. Immunol..

[74] Kook H., Lee S.E., Baik Y.H., Chung S.S., Rhee J.H. (1996). *Vibrio vulnificus* hemolysin dilates rat thoracic aorta by activating guanylate cyclase. Life Sci..

[75] Miyoshi S., Nakazawa H., Kawata K., Tomochika K.-I., Tobe K., Shinoda S. (1998). Characterization of the hemorrhagic reaction caused by *Vibrio vulnificus* metalloprotease, a member of the thermolysin family. Infect. Immun..

[76] Byoung S.K. (2018). The modes of action of MARTX toxin effector domains. Toxins.

[77] Macedo-Ribeiro S., Bode W., Huber R., Quinn-Allen M.A., Kim S.W., Ortel T.L., Bourenkov G.P., Bartunik H.D., Stubbs M.T., Kane W.H. (1999). Crystal structures of the membrane-binding C2 domain of human coagulation factor V. Nature.

[78] Ito N., Philips S.E.V., Stevens C., Ogel Z.B., McPherson M.J., Keen J.N., Yadav K.D.S., Knowles P.F. (1991). Novel thioether bond revealed by a 1.7 A crystal structure of galactose oxidase. Nature.

[79] Linhartová I., Bumba L., Mašín J., Basler M., Osička R., Kamanová J., Procházková K., Adkins I., Hejnová-Holubová J., Sadílková L. (2010). RTX proteins: A highly diverse family secreted by a common mechanism. FEMS Microbiol..

[80] Jorgensen S.E., Mulcahy P.F., Wu G.F., Louis C.F. (1983). Calcium accumulation in human and sheep erythrocytes that is induced by *Escherichia coli* hemolysin. Toxicon.

[81] Diner R.E., Kaul D., Rabines A., Zheng H., Steele J.A., Griffith J.F., Allen A.E. (2021). Pathogenic *Vibrio* species are associated with distinct environmental niches and planktonic taxa in Southern California (USA) aquatic microbiomes. mSystems.

[82] Barrangou R. (2015). The roles of CRISPR-Cas systems in adaptive immunity and beyond. Curr. Opin. Immunol..

[83] Valiente E., Bruhn J.B., Nielsen K.F., Larsen J.L., Roig F.J., Gram L., Amaro C. (2009). *Vibrio vulnificus* produces quorum sensing signals of the AHL-class. FEMS Microbiol. Ecol..

[84] Kim Y.R., Kim B.U., Kim S.Y., Kim C.M., Na H.S., Koh J.T., Choy H.E., Rhee J.H., Lee S.E. (2010). Outer membrane vesicles of *Vibrio vulnificus* deliver cytolysin–hemolysin VvhA into epithelial cells to induce cytotoxicity. Biochem. Biophys. Res. Commun..

[85] Kulp A., Kuehn M.J. (2010). Biological functions and biogenesis of secreted bacterial outer membrane vesicles. Annu. Rev. Microbiol..

[86] MacDonald I.A., Kuehn M.J. (2012). Offense and defense: Microbial membrane vesicles play both ways. Res. Microbiol..

[87] Hampton C.M., Guerrero-Ferreira R.C., Storms R.E., Taylor J.V., Yi H., Gulig P.A., Wright E.R. (2017). The opportunistic pathogen *Vibrio vulnificus* produces outer membrane vesicles in a spatially distinct manner related to capsular polysaccharide. Front. Microbiol..

[88] Zhou J., Ma H., Zhang L. (2023). Mechanisms of Virulence Reprogramming in Bacterial Pathogens. Annu. Rev. Microbiol..

