# Supplementary material for: Comparative Genome Analysis of Piscine Vibrio vulnificus: Virulence-Associated Metabolic Pathways
Source: Microorganisms. 2024 Dec 6;12(12):2518. doi: 10.3390/microorganisms12122518 (PMC11676643; doi:10.3390/microorganisms12122518)
Supplement: Supplementary file 1 [file microorganisms-12-02518-s001.zip › microorganisms-3292585-supplementary.pdf]

Supplementary Figure legend

Figure S1 Venn diagram showed the distribution of shared and unique orthologous gene clusters among *V. vulnificus* samples by OrthoVenn2.

Figure S2 Subsystem category distribution based on RAST annotation server.

Figure S1

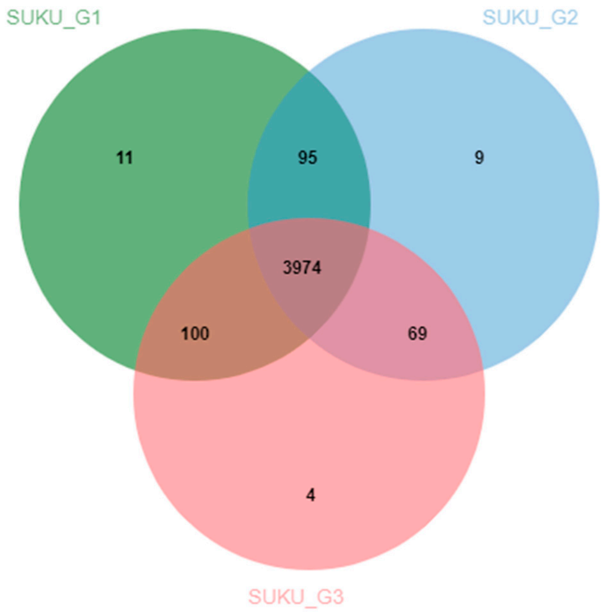

Figure S2

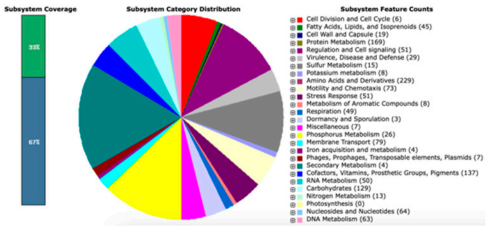

SUKU_G1 chromosome I

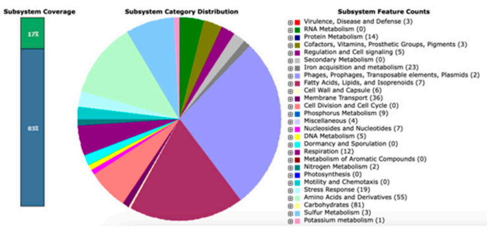

SUKU_G1 chromosome II

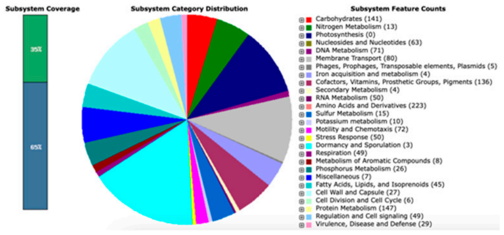

SUKU_G2 chromosome I

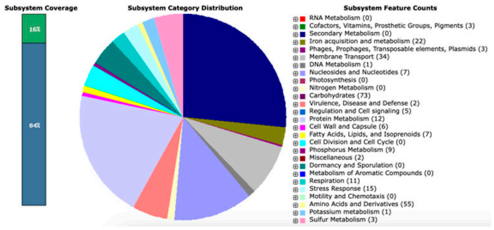

SUKU_G2 chromosome II

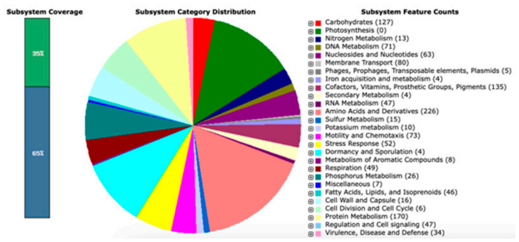

SUKU_G3 chromosome I

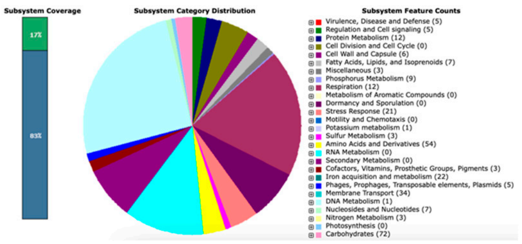

SUKU_G3 chromosome II

Supplementary table legend

Table S1 Reference genomes of *V. vulnificus* and other *Vibrio* species

Table S2 The predicted genes in the 20-kb regions and detailed features from CRISPR prediction

Table S3 Antibiotic resistance profile predicted from CARD

Table S4 ANI value between piscine *V. vulnificus* and strain CMCP6

Table S1

<table>
<tr><th>Species</th><th>Strain</th><th>RefSeq assembly accession</th></tr>
<tr><td rowspan="17">V. vulnificus</td><td>SUKU_G1</td><td rowspan="3">In this study</td></tr>
<tr><td>SUKU_G2</td></tr>
<tr><td>SUKU_G3</td></tr>
<tr><td>FORC_037</td><td>GCF_002204915.1</td></tr>
<tr><td>2142-77</td><td>GCF_009665475.1</td></tr>
<tr><td>Vv180806</td><td>GCF_014107515.1</td></tr>
<tr><td>CECT_4999</td><td>GCF_002215135.1</td></tr>
<tr><td>93U204</td><td>GCF_000746665.1</td></tr>
<tr><td>FORC_054</td><td>GCF_002863725.1</td></tr>
<tr><td>VV2014DJH</td><td>GCF_002850455.1</td></tr>
<tr><td>FORC_053</td><td>GCF_003522555.1</td></tr>
<tr><td>FORC_036</td><td>GCF_002117205.1</td></tr>
<tr><td>2497-87</td><td>GCF_014211935.1</td></tr>
<tr><td>FORC_017</td><td>GCF_001675245.1</td></tr>
<tr><td>FDAARGOS_663</td><td>GCF_008693685.1</td></tr>
<tr><td>FDAARGOS_119</td><td>GCF_001558515.2</td></tr>
<tr><td>FORC_016</td><td>GCF_001653775.1</td></tr>
</table>

	06-2410	GCF_009764095.1
	FORC_009	GCF_001433435.1
	FORC_077	GCF_004319645.1
	CMCP6	GCF_000039765.1
	Env1	GCF_003047125.1
	MO6-24/O	GCF_000186585.1
	ATCC_27562	GCF_002224265.1
	YJ016	GCF_000009745.1
V. alginolyticus	ATCC_33787	GCF_001469735.1
V. harveyi	ZJ0603	GCF_000275705.1
V. parahaemolyticus	ATCC 17802	GCF_001011015.1

Table S2

Location	Function description
SUKU_G1_Chromosome1_2000410_2002827_-	sensor_protein_TorS
SUKU_G1_Chromosome1_2003139_2003624_+	Hypothetical_protein_yecA.,
SUKU_G1_Chromosome1_2003710_2004918_-	Glutathionylspermidine synthase
SUKU_G1_Chromosome1_2004875_2005432_-	DUF1190, [Function unknown]
SUKU_G1_Chromosome1_2005441_2005857_-	Predicted membrane protein, [Function unknown]
SUKU_G1_Chromosome1_2005857_2006897_-	Ion channel
SUKU_G1_Chromosome1_2007003_2008064_-	quinolinate synthase NadA.
SUKU_G1_Chromosome1_2008192_2008974_-	Uncharacterized protein [Function unknown]
SUKU_G1_Chromosome1_2008990_2009535_-	peptidoglycan-associated lipoprotein Pal.
SUKU_G1_Chromosome1_2009567_2010916_-	TolB
SUKU_G1_Chromosome1_2012011_2012451_-	TolR
SUKU_G1_Chromosome1_2012451_2013134_-	TolQ
SUKU_G1_Chromosome1_2013124_2013504_-	Acyl-CoA_thioesterase_YbgC
SUKU_G1_Chromosome1_2013688_2013990_-	YbgE
SUKU_G1_Chromosome1_2013983_2014090_-	outer membrane lipoprotein [Function unknown]
SUKU_G1_Chromosome1_2014103_2015239_-	cytochrome d ubiquinol oxidase subunit II.
SUKU_G1_Chromosome1_2015256_2016842_-	cytochrome bd-I ubiquinol oxidase subunit CydA.
SUKU_G1_Chromosome1_2017305_2018309_-	ruvB, Holliday junction branch migration DNA helicase
SUKU_G1_Chromosome1_2018331_2018945_-	ruvA, Holliday junction branch migration protein
SUKU_G1_Chromosome1_2019016_2020476_-	Methyl-accepting chemotaxis-like domains (chemotaxis sensory transducer)

Table S2 (Continue)

Location	Function description
SUKU_G1_Chromosome2_1240745_1241659_-	Cupin
SUKU_G1_Chromosome2_1241784_1242056_+	nitrate/nitrite transporter NrtS.
SUKU_G1_Chromosome2_1242061_1242963_+	Methyl-accepting chemotaxis-like domains (chemotaxis sensory transducer)
SUKU_G1_Chromosome2_1242999_1243706_-	Lipoate-protein ligase A, LplA
SUKU_G1_Chromosome2_1243780_1244934_-	ARO8
SUKU_G1_Chromosome2_1245048_1245917_+	GyrI-like
SUKU_G1_Chromosome2_1245991_1246806_-	phosphonoacetaldehyde hydrolase; Provisional.
SUKU_G1_Chromosome2_1246897_1248276_-	aspartate aminotransferase family protein.
SUKU_G1_Chromosome2_1248303_1249406_-	2-aminoethylphosphonate--pyruvate transaminase; Provisional.
SUKU_G1_Chromosome2_1249678_1250685_+	phnS2, putative 2-aminoethylphosphonate ABC transporter
SUKU_G1_Chromosome2_1252204_1253310_+	PhnT2, putative 2-aminoethylphosphonate ABC transporter
SUKU_G1_Chromosome2_1253334_1255043_+	PhnU2, putative 2-aminoethylphosphonate ABC transporter
SUKU_G1_Chromosome2_1255053_1255757_+	phnR, phosphonate utilization transcriptional regulator PhnR.
SUKU_G1_Chromosome2_1255877_1256779_+	phn_DUF6, phosphonate utilization associated putative membrane protein.
SUKU_G1_Chromosome2_1256921_1258369_+	Phn_aa_oxid, putative aminophosphonate oxidoreductase
SUKU_G1_Chromosome2_1258485_1259363_+	DNA-binding transcriptional activator GcvA; Provisional.
SUKU_G1_Chromosome2_1259478_1260263_+	APH, Phosphotransferase enzyme family
SUKU_G1_Chromosome2_1260354_1261838_-	MFS_DtpA_like,
SUKU_G1_Chromosome2_1262207_1263577_-	hypothetical_protein
SUKU_G1_Chromosome2_1263762_1264619_+	YdcF-like protein

Table S3

RGI criteria	ARO term	AMR gene family	Drug class	Resistance mechanism	% identity		
					SUKU_G1	SUKU_G2	SUKU_G3
Strict	CRP	RND antibiotic efflux pump	macrolide, fluoroquinolone, penam	antibiotic efflux	95.24	95.24	95.24
Strict	varG	Subclass B1 *V. cholerae* varG beta-lactamase	carbapenem	antibiotic inactivation	83.24	82.71	82.98
Strict	parE	fluoroquinolone resistance parE	fluoroquinolone	antibiotic target alteration	79.3	79.3	79.3
Strict	PBP3	Penicillin-binding protein mutations conferring resistance to beta-lactam antibiotics	cephalosporin, cephamycin, penam	antibiotic target alteration	46.06	46.23	46.06
Strict	adeF	RND antibiotic efflux pump	fluoroquinolone, tetracycline	antibiotic efflux	42.21	42.4	42.21

Table S4

	SUKU_G1	SUKU_G2	SUKU_G3	*V. vulnificus* CMCP6
SUKU_G1	*	98.14	98.19	98.19
SUKU_G2	98.18	*	98.28	98.2
SUKU_G3	98.25	98.25	*	98.38
V. vulnificus CMCP6	98.14	98.09	98.33	*

The asterisk (*) represents the ANI value compared to itself.
